# Identification of sepsis-associated mitochondrial genes through RNA and single-cell sequencing approaches

**DOI:** 10.1186/s12920-024-01891-x

**Published:** 2024-05-03

**Authors:** Shilin Li, Xiang Li, Sishi Jiang, Chenglin Wang, Yingchun Hu

**Affiliations:** Emergency Medicine Department, The Affiliated Hospital of Southwest Medical University, 25 Taiping Street, Jiangyang District, Luzhou, Sichuan China

**Keywords:** Sepsis, Mitochondria-associated genes(MiAGs), scRNA-seq, Prognosis

## Abstract

**Background:**

Sepsis ranks among the most formidable clinical challenges, characterized by exorbitant treatment costs and substantial demands on healthcare resources. Mitochondrial dysfunction emerges as a pivotal risk factor in the pathogenesis of sepsis, underscoring the imperative to identify mitochondrial-related biomarkers. Such biomarkers are crucial for enhancing the accuracy of sepsis diagnostics and prognostication.

**Methods:**

In this study, adhering to the SEPSIS 3.0 criteria, we collected peripheral blood within 24 h of admission from 20 sepsis patients at the ICU of the Southwest Medical University Affiliated Hospital and 10 healthy volunteers as a control group for RNA-seq. The RNA-seq data were utilized to identify differentially expressed RNAs. Concurrently, mitochondrial-associated genes (MiAGs) were retrieved from the MitoCarta3.0 database. The differentially expressed genes were intersected with MiAGs. The intersected genes were then subjected to GO (Gene Ontology), and KEGG (Kyoto Encyclopedia of Genes and Genomes) analyses and core genes were filtered using the PPI (Protein-Protein Interaction) network. Subsequently, relevant sepsis datasets (GSE65682, GSE28750, GSE54514, GSE67652, GSE69528, GSE95233) were downloaded from the GEO (Gene Expression Omnibus) database to perform bioinformatic validation of these core genes. Survival analysis was conducted to assess the prognostic value of the core genes, while ROC (Receiver Operating Characteristic) curves determined their diagnostic value, and a meta-analysis confirmed the accuracy of the RNA-seq data. Finally, we collected 5 blood samples (2 normal controls (NC); 2 sepsis; 1 SIRS (Systemic Inflammatory Response Syndrome), and used single-cell sequencing to assess the expression levels of the core genes in the different blood cell types.

**Results:**

Integrating high-throughput sequencing with bioinformatics, this study identified two mitochondrial genes (*COX7B, NDUFA4*) closely linked with sepsis prognosis. Survival analysis demonstrated that patients with lower expression levels of *COX7B* and *NDUFA4* exhibited a higher day survival rate over 28 days, inversely correlating with sepsis mortality. ROC curves highlighted the significant sensitivity and specificity of both genes, with AUC values of 0.985 for *COX7B* and 0.988 for *NDUFA4*, respectively. Meta-analysis indicated significant overexpression of *COX7B* and *NDUFA4* in the sepsis group in contrast to the normal group (*P* < 0.01). Additionally, single-cell RNA sequencing revealed predominant expression of these core genes in monocytes-macrophages, T cells, and B cells.

**Conclusion:**

The mitochondrial-associated genes (MiAGs) *COX7B* and *NDUFA4* are intimately linked with the prognosis of sepsis, offering potential guidance for research into the mechanisms underlying sepsis.

## Introduction

Sepsis represents a critical condition and poses a significant threat to life marked by an imbalanced reaction of the body to infection, which results in the failure of multiple organs (MOF) [1]. Current research has unveiled that the pathophysiological mechanisms of this disease primarily encompass mitochondrial metabolic disorders, alterations in molecular expression levels, and an increase in apoptosis [2]. Under septic conditions, mitochondrial activity becomes imbalanced, with significant morphological changes and functional suppression observed. Electron microscopy has revealed that mitochondria isolated from vital organs such as the liver, kidneys, heart, lungs, and brain of sepsis animal models exhibit ultrastructural damage. This includes mitochondrial swelling, cristae disappearance, formation of mitochondrial vacuoles, matrix degradation, and membrane rupture, alongside phenomena like mitochondrial deformation and shrinkage [3]. Concurrently, mitochondrial physiological integrity is compromised in septic states, evidenced by oxidative stress induction, calcium ion hyperaccumulation, and diminution of biological efficacies [4, 5]. Given their pivotal involvement in cellular proliferation and demise, mitochondria emerge as quintessential pharmacological nexuses in the therapeutic landscape of sepsis [6].

Contemporary discourse posits that the recalibration of the oxidative and antioxidative milieu within the mitochondria, potentially through the augmentation of their biosynthetic capacities, may constitute a cornerstone in the enhanced management of sepsis sufferers. Evidence has illuminated that various antioxidants or molecular entities could ostensibly ameliorate the prognostic outlook for individuals afflicted with sepsis. Among the therapeutic interventions that have undergone evaluation in human subjects, ascorbic acid, nitric oxide synthase (NOS) inhibitors, and melatonin stand out. Ascorbic acid, manifesting as the oxidized-reduced variant of vitamin C and an intrinsic antioxidant, has successfully navigated Phase I safety evaluations in septic cohorts. Ketanserin, an inhibitor of NOS, has been corroborated to confer benefits upon septic patients, notably through the amelioration of microcirculatory perfusion. Melatonin, synthesized within the cerebral pineal gland, exerts anti-inflammatory and anti-apoptotic influences, operates as a scavenger for reactive oxygen and nitrogen species, and has been substantiated to mitigate inflammatory and oxidative stress markers in models of human endotoxemia [7]. Contrastingly, endeavors targeting mitochondrial biosynthesis have been met with restrained advancement, attributed to the intricate nature of their biosynthetic pathways. This realm encompasses several putative therapeutic targets integral to mitochondrial biogenesis, such as PGC-1α, Tfam, and NRF-1. Additionally, Mitogen-Activated Protein Kinase Kinase 3 (MKK3) has been unveiled to serve a critical function in the mitochondrial biosynthetic process within sepsis murine models [8].

Thus, it becomes evident that mitochondria act as a key factor in the onset and progression of sepsis. There has been a notable increase in research focusing on the role of mitochondria within the physiological and pharmacological pathways of sepsis; however, studies investigating biomarkers associated with mitochondrial genes for identifying and predicting the outcome of sepsis remain relatively scarce. Targeted treatment of sepsis based on mitochondrial-related gene biomarkers heralds a new direction in sepsis research and therapy. Consequently, we employed high-throughput sequencing technology to conduct RNA sequencing on peripheral blood cells from 20 sepsis patients and 10 healthy individuals. Through bioinformatics methods, Mitochondria-associated genes (MiAGs) intimately linked to the prognosis of sepsis were identified, and *single-cell sequencing was used to evaluate the cell specific expression levels of the MiAGs*. This provides guidance for research into sepsis-related mechanisms and offers new insights for the clinical management of sepsis.

## Methods

### Data collection

This experiment was conducted from January 2019 to December 2019 in the ICU of Southwest Medical University Affiliated Hospital in Luzhou City, Sichuan Province, China, where peripheral blood samples were collected from 20 sepsis patients and 10 healthy individuals. The collected peripheral blood samples were utilized for RNA-seq. The RNA-seq data were employed to screen for genes differentially expressed between the sepsis patients and the healthy control group. Inclusion criteria for sepsis cases were: (1) meeting the diagnostic criteria for sepsis; (2) no clear history of acute or chronic liver diseases; (3) no severe infectious diseases or immune dysfunction history; (4) first-time diagnosis and treatment; (5) no metabolic disease history. Exclusion criteria included: (1) significant organ dysfunction; (2) concurrent malignant tumors; (3) immunosuppressive therapy within the last 3 months; (4) severe congenital diseases or malformations. All participants signed informed consent, and the study protocol was approved by the Ethics Committee of the Affiliated Hospital of Southwest Medical University (Ethics number: ky2018029), with a clinical trial registration number of ChiCTR1900021261. The work involving human subjects adhered to the principles outlined in the Declaration of Helsinki [9].

### PAX sample preprocessing

The collection of whole blood constitutes the initial step for multiple molecular assays aimed at studying intracellular RNA. The PAXgene® Blood RNA System (BD Biosciences) is comprised of a blood collection tube (PAXgene® Blood RNA Tube) and a nucleic acid purification kit (PAXgene® Blood RNA Kit). The PAXgene® Blood RNA Vessel harbors a concoction tailored to perpetuate the intrinsic gene transcription panorama by mitigating RNA disintegration ex vivo and attenuating gene elicitation. In synergy with the PAXgene® Blood RNA Apparatus, this vessel secures meticulous discernment and enumeration of gene articulations [10]. Prior to specimen collection, it was ensured that the PAXgene® Blood RNA Tube was at a temperature between 18 °C and 25 °C and correctly labeled with patient information. A standard venipuncture procedure of the donor was performed, drawing blood into the PAXgene® Blood RNA Tube using the blood collection set and holder. Immediately after blood collection, the PAXgene® Blood RNA Tube was gently inverted 8–10 times and stored it at 18–25 °C for three days before proceeding with total RNA purification.

#### RNA sequencing

Total RNA was extracted from blood samples utilizing the Trizol method (Invitrogen, Carlsbad, CA, USA) and quantitatively analyzed via the Agilent 2100 bioanalyzer (Thermo Fisher Scientific, MA, USA). In accordance with the manufacturer’s protocol, the initial phase involved the depletion of ribosomal RNA (rRNA) via target-specific oligonucleotides and Ribonuclease H reagents. Following SPRI bead purification, the RNA was fragmented into small pieces at elevated temperatures in the presence of divalent cations. The cleaved RNA fragments were then reverse transcribed into first-strand cDNA using reverse transcriptase and random primers, followed by synthesis of the second-strand cDNA using DNA Polymerase I and RNase H. The quality and quantity of the library were assessed through two methodologies: fragment size distribution was examined using the Agilent 2100 bioanalyzer, and library quantification was performed via quantitative PCR (qPCR) with TaqMan Probe. Qualified libraries underwent paired-end sequencing on the BGISEQ-500/MGISEQ-2000 platform (BGI-Shenzhen, China). Sequencing raw data (lncRNA/mRNA, miRNA) was filtered using the filtration software SOAPnuke (https://github.com/BGI-flexlab/SOAPnuke), with filtered clean reads saved in FASTQ format [11].

### Identification of differentially expressed genes

The RNA-seq raw data were processed utilizing the online analysis platform IDEP2.0 (Integrated Differential Expression & Pathway Analysis) (http://bioinformatics.sdstate.edu/idep/) [12] Initially, the data were uploaded to the IDEP2.0 platform’s LoadData interface, where the data type was specified as Read counts data. Upon successful upload, the data underwent log transformation and normalization, with the processed results accessible in the preprocessing interface. This interface facilitated the assessment of sample uniformity through box plots and density distribution graphs. Subsequently, the Principal Component Analysis (PCA) interface enabled the visualization of intergroup differences. For the analysis of differential expression, the DEG2 page was utilized, employing criteria of Fold Change (FC) ≥ 2 and False Discovery Rate (FDR) < 0.05. Differential expression results were then visualized using volcano plots generated with the ggplot2 package in R, providing a graphical representation of the differentially expressed genes (DEGs). This comprehensive analytical approach within the IDEP2.0 platform ensured thorough exploration and visualization of RNA-seq data, facilitating the identification of biologically significant DEGs under stringent statistical thresholds.

### Mitochondrial gene selection

MitoCarta3.0, a compendium encompassing 1136 human and 1140 mouse genes coding for proteins robustly supported for mitochondrial placement, now integrates annotations for sub-mitochondrial locales and biochemical pathways [13]. Thus, the MitoCarta 3.0 database was utilized for mitochondrial gene selection. Entering “mitochondria” in the search bar of the MitoCarta3.0 database, selecting “Gene Product,” and downloading genes related to human mitochondria facilitated the identification of mitochondria-associated genes tightly linked to sepsis by overlapping genes showing variance with those associated with mitochondria.

### GO analysis

GO analysis provides a method for categorizing genes into three categories: biological process (BP), cellular component (CC), and molecular function (MF) [14]. To further explore whether intersected genes are involved in specific functions, the “clusterProfiler” package in R was employed for GO analysis, with *p* < 0.05 considered statistically significant.

### Kyoto encyclopedia of genes and genomes (KEGG) analysis

The KEGG database is a comprehensive repository for the methodical examination of gene roles, bridging genomic data with insights into their functionality, encompassing metabolic pathways, hierarchical classification databases, and genome databases [14]. Signal pathways form the essence of basic scientific research, setting the stage for subsequent pathway analysis. The “clusterProfiler，ggplot2” package in R version 4.2.1 was applied for enrichment analysis of intersected genes, visualizing the results with a significance range set at *p* < 0.05.

### Protein-protein interaction network

To further refine potential core genes, intersected genes were submitted to the STRING database (https://cn.string-db.org/) [15]. Protein interaction data were exported to Cytoscape (version 3.4.0), where the cytoHubba plugin was employed for ranking nodes within the network based on their network features. CytoHubba provides 11 topological analysis methods, including degree, edge percolation component, maximum neighborhood component, maximum neighborhood component density, maximal clique centrality (MCC), and six centrality measures (Bottleneck, Eccentricity, Closeness, Radiality, Betweenness, and Stress) [16]. Among these methods, MCC has been demonstrated to offer superior performance in predicting essential proteins within the PPI network. Therefore, this algorithm was utilized to identify core genes situated within the network.

### Survival analysis

Survival analysis, a staple in biomedical research [17], was conducted to investigate the relationship between potential mitochondrial core genes identified through PPI analysis and the prognosis of sepsis patients. Public dataset GSE65682, submitted by Scicluna BP et al. in 2015 and comprising gene data related to over 479 sepsis patients and their 28-day prognosis, was retrieved from the GEO database (https://www.ncbi.nlm.nih.gov/geo/) [18]. Data extraction and analysis were performed by Graphpad Prism 9.0, with the logrank test for statistical evaluation, setting *p* < 0.05 as the threshold for significance.

### Receiver operating characteristic (ROC) curve

The area under the ROC curve (AUC) is utilized to assess the model’s efficacy in distinguishing between patients with and without a disease, with higher AUC scores indicating superior diagnostic accuracy [19]. This study utilized dataset GSE95233 from the GEO database, comparing septic shock patients and healthy volunteers. Septic shock patients had samples collected at admission and again on either day 2 or day 3. The admission samples of septic shock patients were compared with those of healthy volunteers, analyzing gene expression regulation between the two time points based on a 28-day survival rate. The ROC curves were analyzed using MedCalc software to evaluate the diagnostic accuracy of core genes for sepsis.

### Meta-analysis

To further assess the expression of sepsis-associated mitochondrial genes across different populations and validate our findings with public data, we searched and retrieved sepsis datasets GSE28750, GSE54514, GSE67652, GSE69528, GSE95233 from the GEO database, dividing the data into sepsis (SEPSIS) and normal control (NC) groups. A meta-analysis of the core genes’ expression levels was conducted, culminating in the construction of forest plots [20].

### Single-cell sequencing

Single-cell genomics reveals the cellular localization of target genes within tissues and their functions and characteristics at different stages and aspects [21]. This study employed the 10x Genomics single-cell sequencing technique to investigate the cell lineage positioning of target genes. Five blood samples were collected (NC = 2; Sepsis = 2; SIRS = 1;) and combined. PBMCs were isolated via density gradient centrifugation for 10x Genomics sequencing, producing raw reads in FASTQ format, which were quality analyzed using CellRanger software. Data underwent further quality control with the Seurat software package, employing PCA for dimension reduction and tSNE for visualization. Additionally, the FindMarkers function was used to identify gene biomarkers. The cellular localization of specific target genes aids in selecting specific cell lineages for future in vitro functional studies.

## Results

### Demographics and clinical characteristics

This study encompassed 20 sepsis patients and 10 healthy individuals. Patient demographics such as gender, age, 28-day survival status, and clinical markers of organ function including Alanine Aminotransferase (ALT), Aspartate Aminotransferase (AST), Total Bilirubin (TBIL), Indirect Bilirubin (I-Bil), Creatinine (Crea), Urea, Total White Blood Cell (WBC) count, Neutrophil count, Monocytes (MONO), and Lymphocyte (LY) were collected. Unpaired T-tests were utilized for statistical analysis. The results indicated statistical differences among the sepsis cohort and the comparison group in AST, TBIL, Crea, Urea, and WBC (*p* < 0.05), suggesting impaired organ function in sepsis patients (Table 1).

**Table 1:** Demographic and clinical data of subjects (m ± sd). Gender, 28-day Finale(S: survivor, D: death), age, ALT, AST, TBIL, I-Bil, (Crea), Urea, WBC, neutrophil count, MONO, LY.

Clinic items	Sepsis(*n* = 20)	NC (*n* = 10)	*p*
Gender (F/M) Day Finale (S/D)	13/7 15/5	6/4 10/0	- -
Age(years)	58.45 ± 16.87	53.50 ± 7.66	0.069
ALT(U/L)	96.11 ± 190.73	20.94 ± 6.44	0.067
AST(U/L)	159.42 ± 288.87	22.18 ± 4.53	0.020
TBIL(umol/L)	33.72 ± 40.15	16.79 ± 6.37	0.039
I-Bil(umol/L)	11.58 ± 11.10	11.55 ± 4.36	0.097
Crea(umol/L)	128.86 ± 136.04	63.75 ± 9.30	0.001
Urea(mmol/L)	13.37 ± 14.80	5.05 ± 1.49	0.006
WBC(10^9/L)	13.77 ± 7.08	6.88 ± 1.85	0.019
NEUT(10^9/L)	14.82 ± 13.55	4.13 ± 1.51	0.050
MONO(10^9/L)	0.86 ± 1.21	0.44 ± 0.18	0.110
LY(10^9/L)	1.12 ± 1.62	2.02 ± 0.57	0.232

### Identification of sepsis mitochondrial-associated genes

**Fig. 1:**
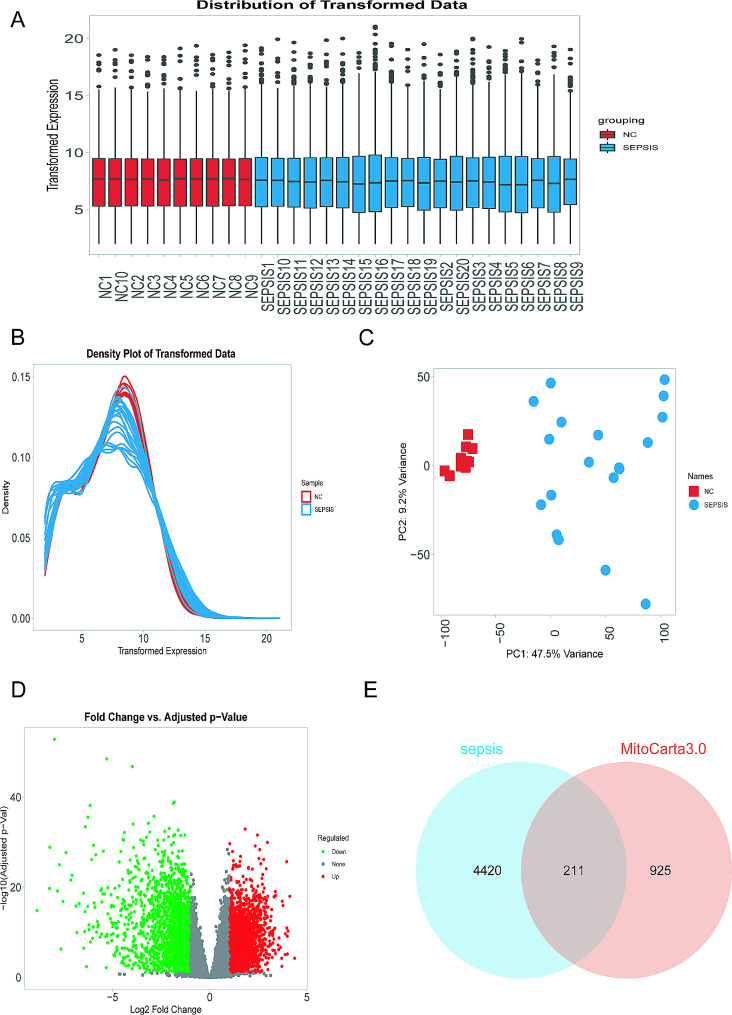
Selection of Sepsis Differentially Expressed Genes and Mitochondrial-Related Genes. (**A-B**) Box plots and density distribution plots illustrate the homogeneity and comparability of data distributions after normalization for two datasets. (**C**) Principal component analysis demonstrates significant differences between our sepsis and normal groups. (**D**) Volcano plot of differentially expressed genes, with red indicating 2419 genes upregulated and green indicating 2212 genes downregulated in the sepsis group. (**E**) Venn diagram depicting the intersection of sepsis differentially expressed genes (light blue, *n* = 4631) and mitochondrial genes (pink, *n* = 1136), yielding 211 intersecting genes

Quality control analysis was conducted on the sequenced mRNA, revealing a distinct partition of all samples into two cohorts. Box plots and density distribution graphs demonstrated the homogeneity and comparability of the two datasets, elucidating the distribution of normalized data (Fig. 1A-B). Principal component analysis depicted pronounced disparities between our sepsis and normal cohorts (Fig. 1C). Differential analysis was employed on the sequenced mRNA from both cohorts, selecting for 4631 Differentially Expressed mRNAs (DEmRNAs) with an absolute fold change (FC) ≥ 2 and false discovery rate (FDR) < 0.05, comprising 2419 upregulated and 2212 downregulated transcripts (Fig. 1D). A total of 1136 human mitochondrial genes were sourced from the MitoCarta 3.0 database. Intersection of human mitochondrial genes with the 4631 genes exhibiting differential expression in sepsis identified 211 mitochondrial-associated genes closely implicated in sepsis (Fig. 1E).

### GO enrichment analysis

**Fig. 2:**
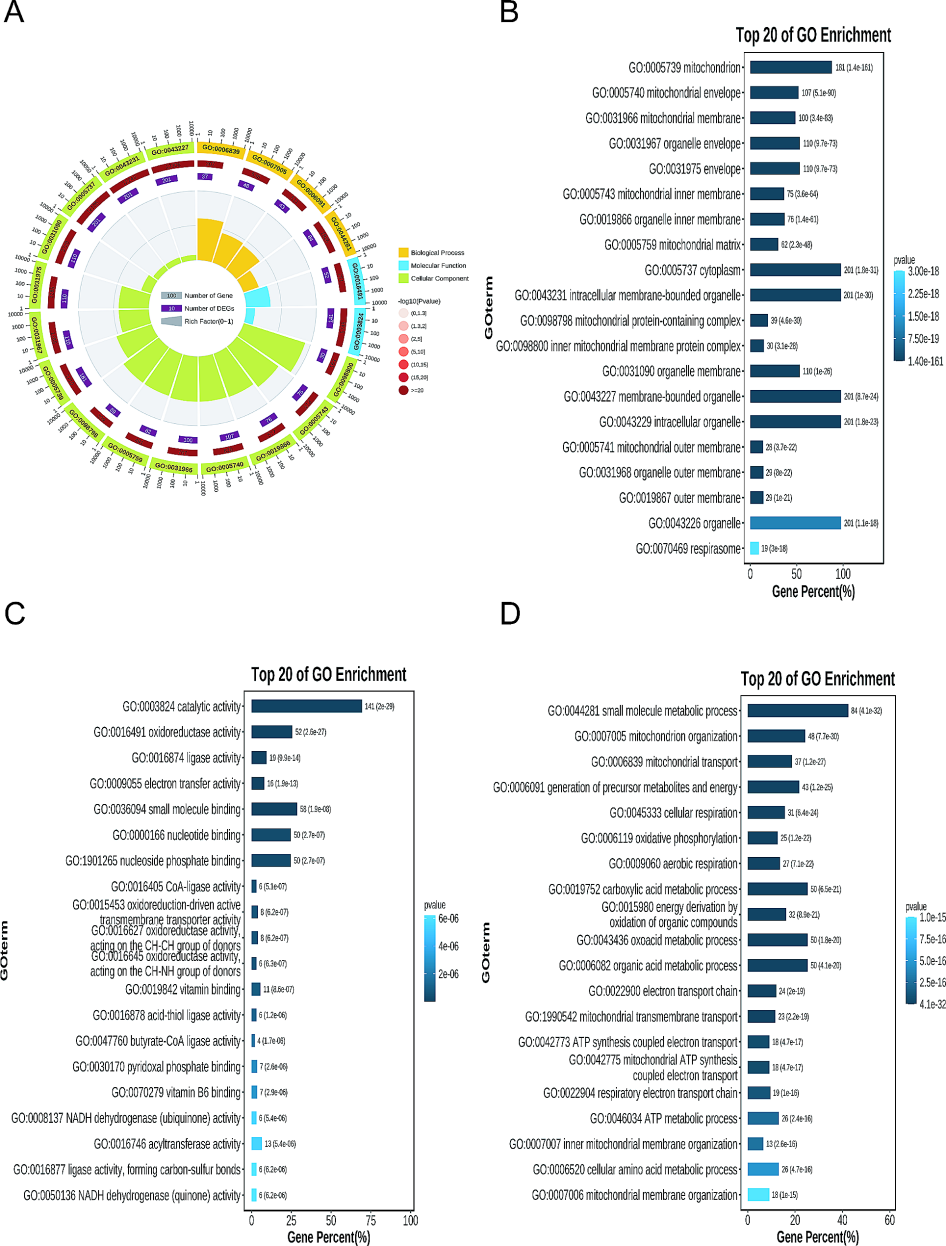
Gene ontology enrichment analysis. (**A**) GO analysis reveals 4 biological process, 14 cellular component, and 2 molecular function categories. (**B**) The cellular components of crossover genes are mainly associated with intracellular membrane-bound organelles, especially mitochondria. (**C**) Molecular function enrichment of intersection genes primarily involves catalytic activity and small molecule binding. (**D**) Biological processes associated with intersection genes mainly include small molecule metabolic process and organic acid metabolic process

GO enrichment analysis revealed that among the top 20 entries, 4 were associated with biological processes, 14 with cellular components, and 2 with molecular functions (Fig. 2A). Crossover genes are mainly involved in cellular components as intracellular membrane-bound organelles, especially mitochondrion (Fig. 2B), with molecular function enrichment mainly involving catalytic activity and small molecule binding (Fig. 2C). Furthermore, the biological processes related to intersected genes mainly encompass small molecule metabolic process and organic acid metabolic process (Fig. 2D).

### KEGG analysis

**Fig. 3:**
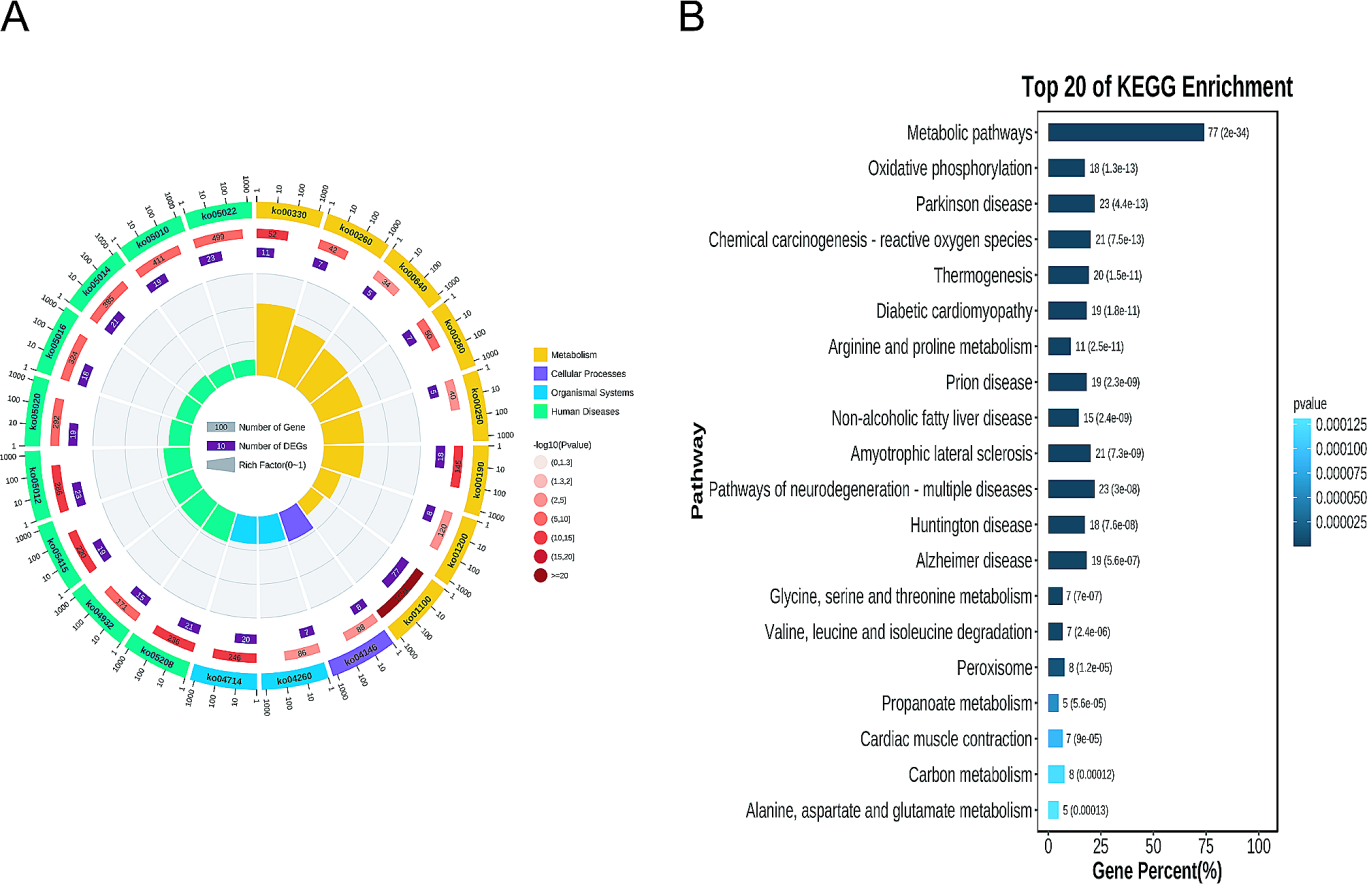
KEGG Enrichment Pathways. (**A**) KEGG analysis identified 20 noteworthy pathways, including 8 metabolism, 1 cellular process, 2 organismal systems, and 9 human diseases. (**B**) Enriched pathways in the intersection genes include Metabolic pathways, Pathways of neurodegeneration-multiple diseases, etc.

KEGG analysis identified 20 significant pathways, including 8 metabolic pathways, 1 cellular process, 2 organismal systems, and 9 human diseases (Fig. 3A). Among the most significant pathways for intersected genes were Metabolic pathways and Pathways of neurodegeneration - multiple diseases (Fig. 3B).

### PPI network

**Fig. 4:**
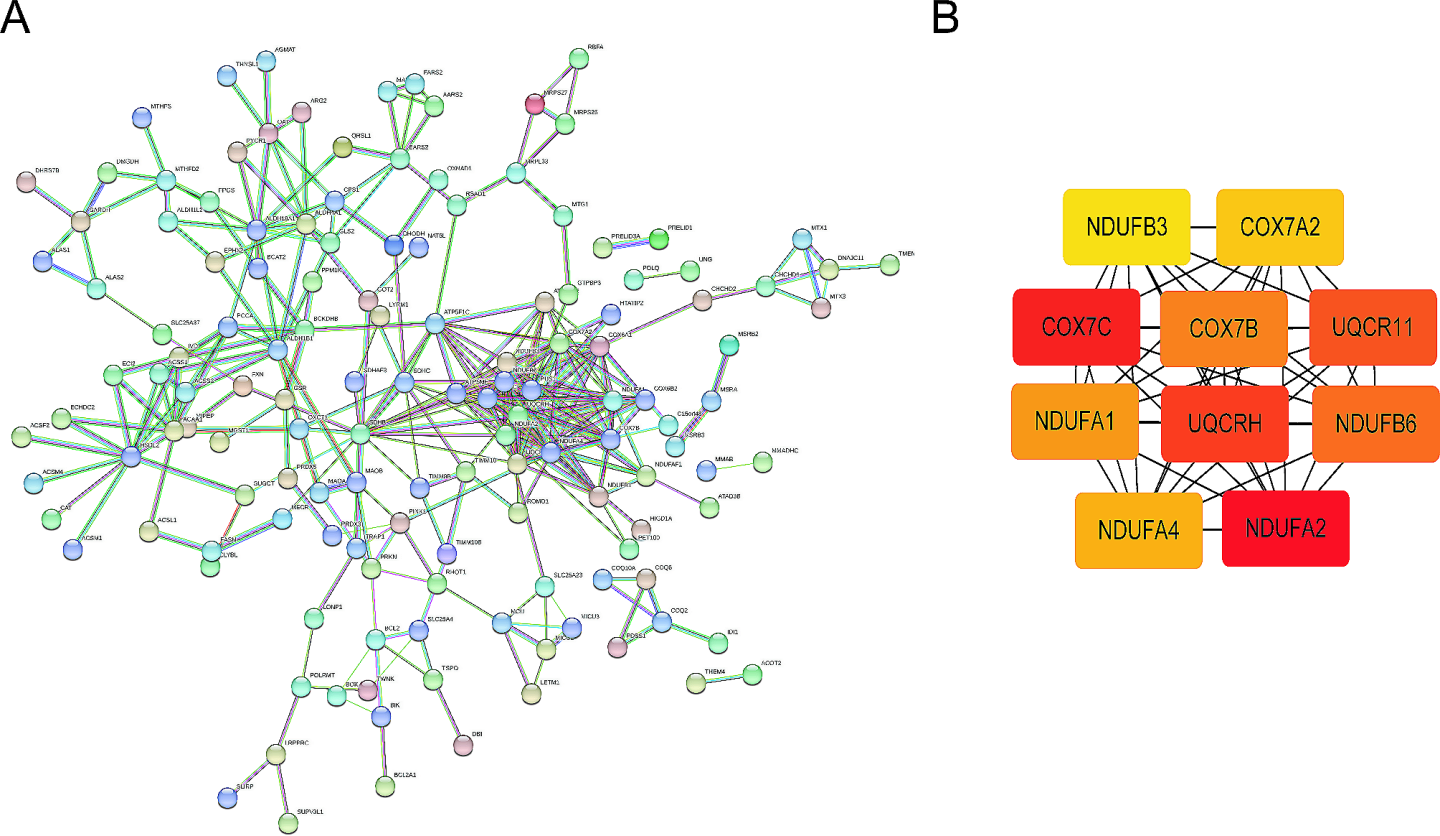
Protein-Protein Interaction (PPI) Network. (**A**) Protein interaction network diagram based on STRING database, where more connecting lines between proteins indicate closer protein-protein interactions. (**B**) The top 10 most central genes obtained from Cytoscape analysis include *NDUFA2, COX7C, UQCRH, UQCR11, NDUFB6, COX7B, NDUFA1, NDUFA4, COX7A2*, and *NDUFB3.*

A PPI network of intersected genes was constructed (Fig. 4A). By importing results into Cytoscape and employing the cytohubba plugin’s MCC algorithm, 10 mitochondrial genes closely related to sepsis were identified, including *NDUFA2, COX7C, UQCRH, UQCR11, NDUFB6, COX7B, NDUFA1, NDUFA4, COX7A2*, and *NDUFB3* (Fig. 4B).

### Discovery of mitochondrial genes associated with diagnosis and prognosis of sepsis

**Fig. 5:**
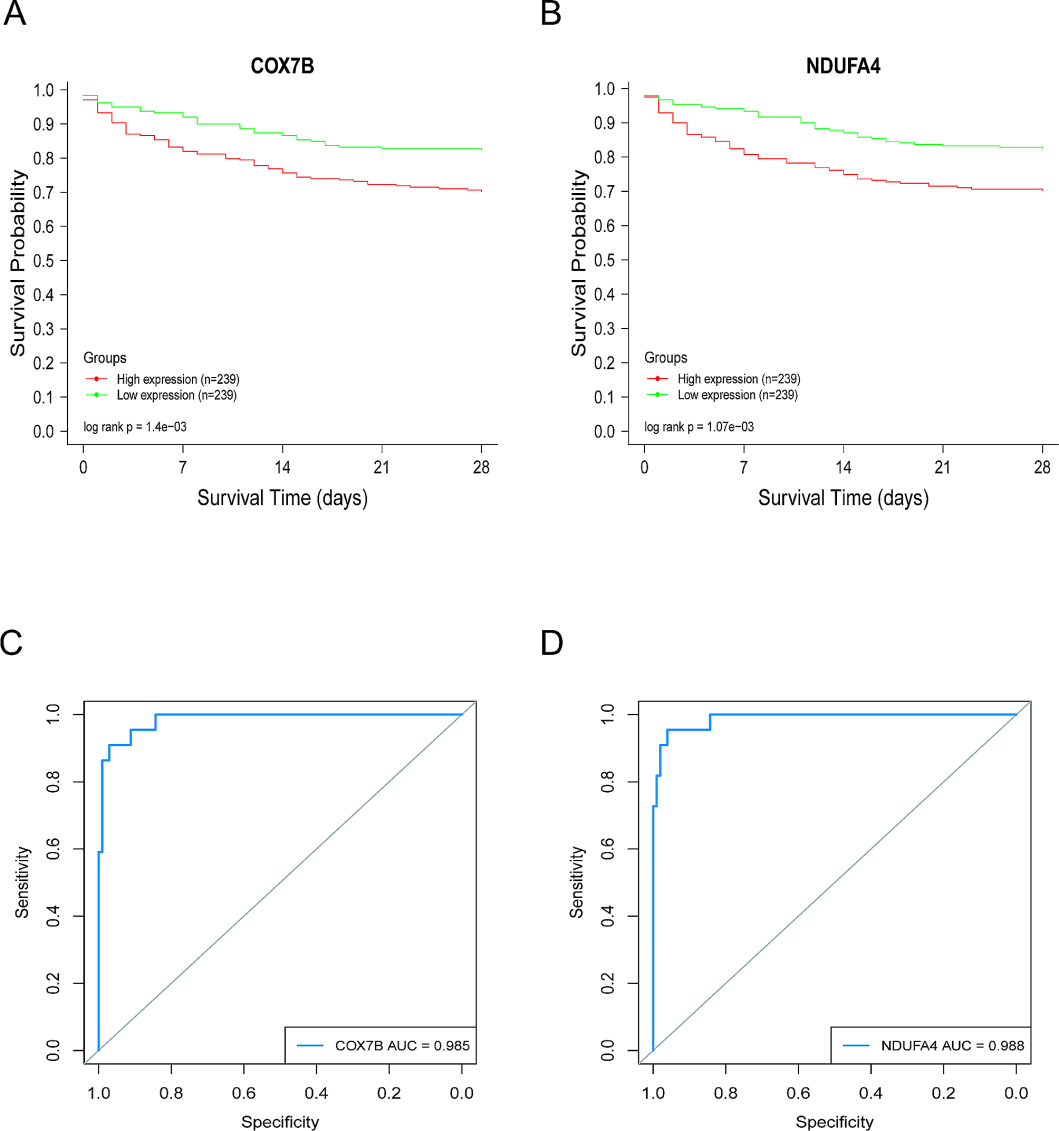
Survival Analysis and ROC Curve. Based on the GSE65682 dataset, all sepsis-specific genes were divided into two groups according to their expression levels. The red line represents the high-expression group (*n* = 239), while the green line represents the low-expression group (*n* = 239). The horizontal axis represents the recorded time, and the vertical axis represents the instantaneous survival rate. (**A-B**) Patients with low expression of *COX7B* and *NDUFA4* exhibit higher 28-day survival rates compared to the high-expression group, showing a negative correlation with sepsis prognosis. The differences are statistically significant (*P* < 0.05). (**C-D**) ROC curve analysis based on the GSE95233 dataset reveals that *COX7B* and *NDUFA4* exhibit high sensitivity and specificity, with AUC values of 0.985 for *COX7B* and 0.988 for *NDUFA4*

Survival analysis based on the dataset GSE65682 revealed a significant correlation between the expression levels of *COX7B* and *NDUFA4* and the 28-day survival rate of patients. Patients with low expression of *COX7B* and *NDUFA4* exhibited higher 28-day survival rates compared to the high-expression group, showing a negative correlation with the survival rate of sepsis patients, with statistically significant differences (*P* < 0.05) (Fig. 5A-B). This finding suggests that these two mitochondrial genes are closely associated with the prognosis of sepsis patients, and their expression levels may become a new focus in sepsis research. Additionally, ROC curve analysis based on the dataset GSE95233 from the GEO database demonstrated that *COX7B* and *NDUFA4* exhibit high sensitivity and specificity, with AUC values of 0.985 and 0.988, respectively (Fig. 5C-D). Therefore, these findings provide valuable insights for the development of mitochondrial-targeted therapy for septicemia.

### Meta-analysis

**Fig. 6:**
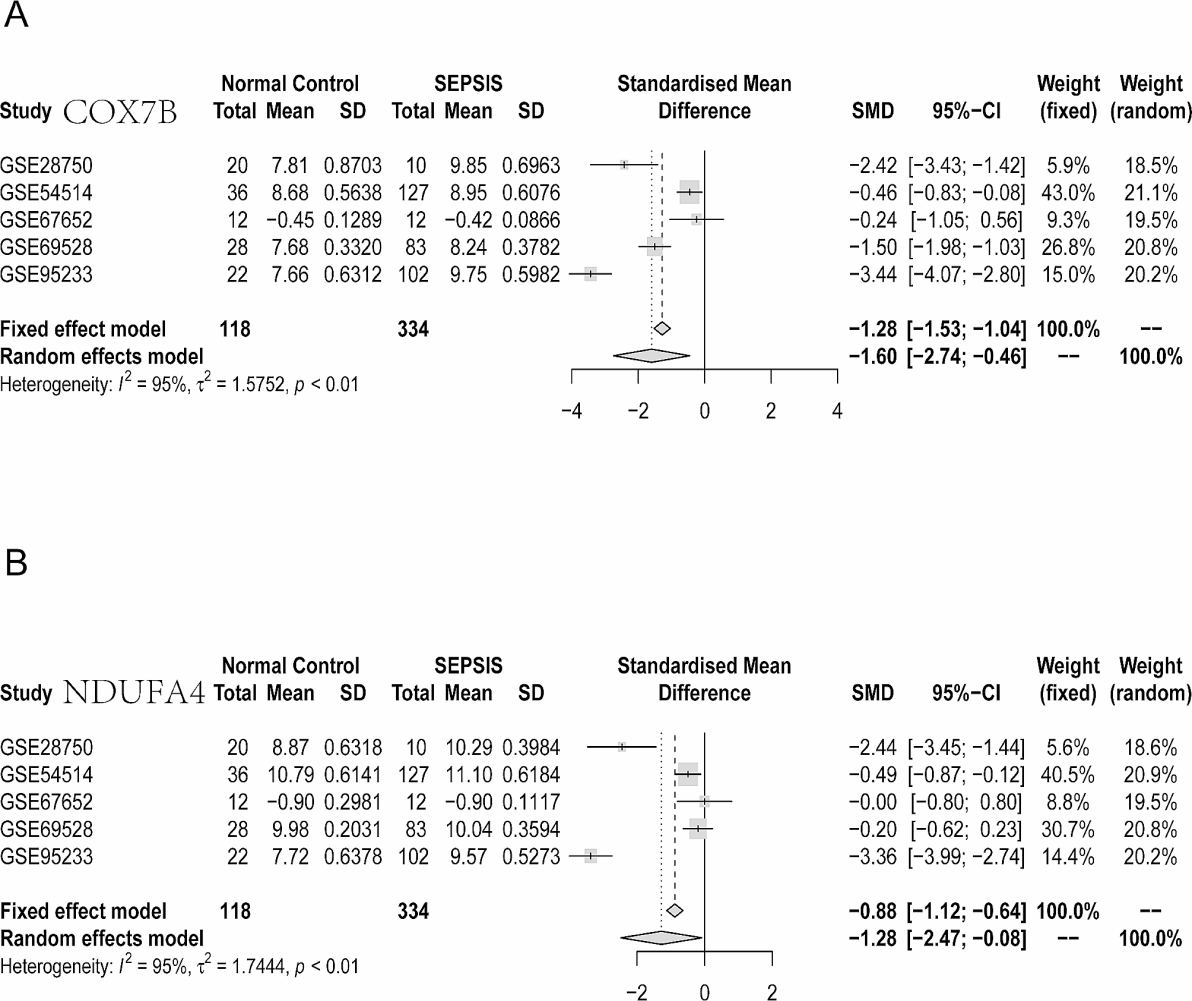
Meta-Analysis. (**A-B**) Meta-analysis based on the GSE28750, GSE54514, GSE67652, and GSE69528 datasets reveals that *COX7B* and *NDUFA4* are upregulated in the septic group compared to the normal group, with statistically significant differences (*P* < 0.01)

Leveraging sepsis datasets from the GEO public database(GSE28750, GSE54514, GSE67652, GSE69528, GSE95233), a transcriptional-level meta-analysis was performed on *COX7B* and *NDUFA4*. This analysis revealed that both *COX7B* and *NDUFA4* exhibit higher expression levels in the sepsis group compared to the normal control group, with this difference being remarkably different (*P* < 0.01) (Fig. 6A-B), indicating their potential significance in sepsis research. For detailed information on the GEO datasets included in the study, refer to Table 2.

**Table 2:** Survival analysis was conducted using the GSE65682 dataset from the GEO database, while meta-analysis utilized the GSE28750, GSE54514, GSE67652, and GSE69528 datasets. ROC curve analysis was performed using the GSE95233.

GSE datasets	Organism	Platform	Number of samples
GSE65682	Whole Blood of Human	GPL13667	802
GSE28750	Whole Blood of Human	GPL570	41
GSE54514	Whole Blood of Human	GPL6947	163
GSE67652	Whole Blood of Human	GPL16699	24
GSE69528	Whole Blood of Human	GPL10558	138
GSE95233	Whole Blood of Human	GPL570	124

#### Single-cell sequencing analysis

**Fig. 7:**
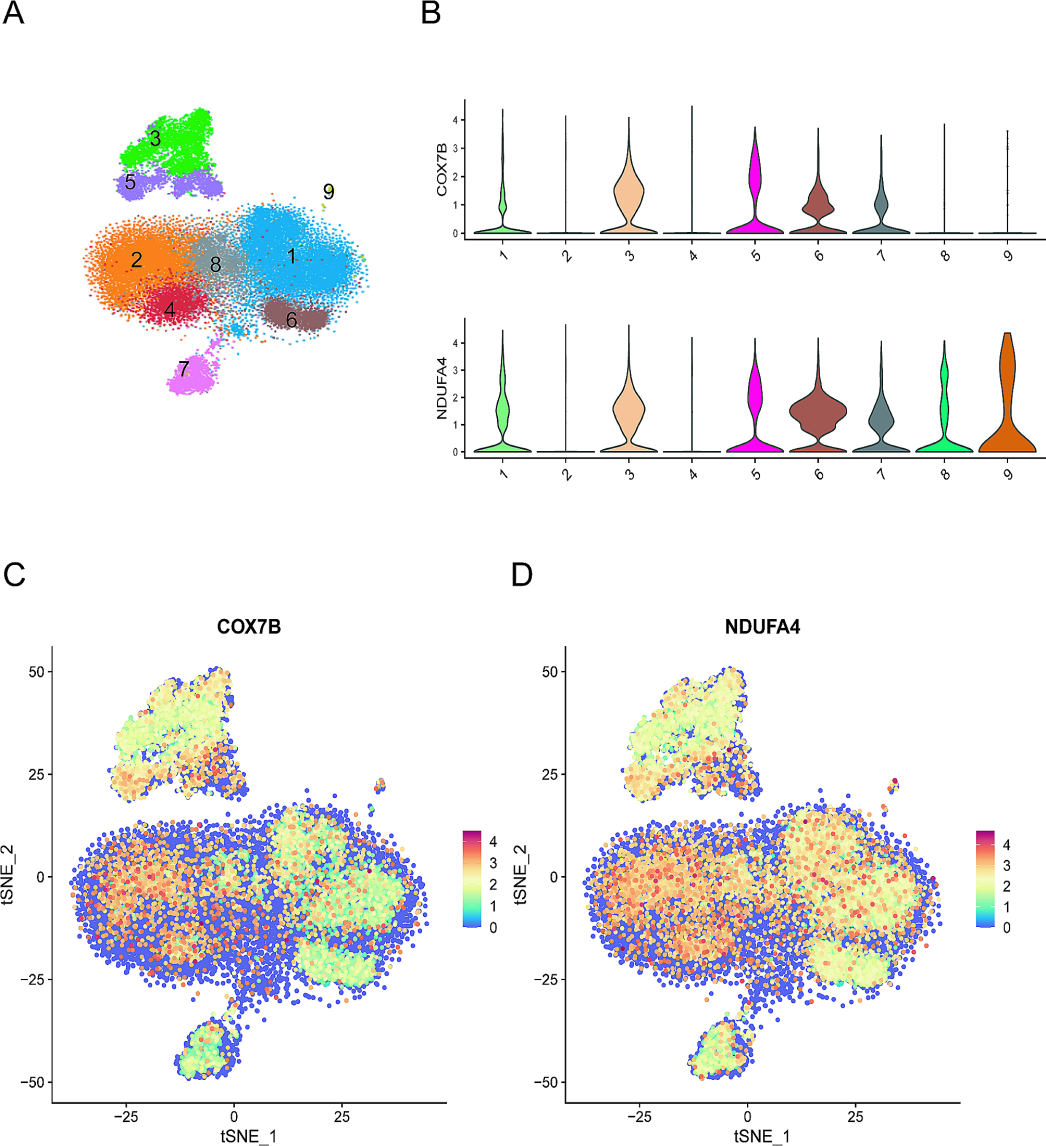
Single-cell Sequencing Localization. (**A-B**) Following hierarchical clustering of mixed samples, cells are divided into 9 clusters. Cell types identified by marker genes include B cells, NK cells, T cells, platelets, and monocyte-macrophages. Clusters 3 and 5 represent monocyte-macrophages, cluster 4 represents NK cells, clusters 1, 2, 6, and 8 represent T cells, cluster 7 represents B cells, and cluster 9 represents platelets. (**C-D**) Core genes *COX7B* and *NDUFA4* are predominantly expressed in monocyte-macrophages, T cells, and B cells

In this study, a total of five single-cell transcriptome sequencing samples were completed. After the exclusion of doublets, multicellular aggregates, and apoptotic cells, the number of viable cells qualified for analysis ranged from 6,000 to 12,000. Subsequent to hierarchical clustering, cells were divided into nine distinct groups. Identification of marker genes revealed the cell types to include B cells, NK cells, T cells, platelets, and monocyte-macrophages. Specifically, clusters 3 and 5 were identified as monocyte-macrophages, cluster 4 as NK cells, clusters 1, 2, 6, and 8 as T cells, cluster 7 as B cells, and cluster 9 as platelets (Fig. 7A). Assessment of single-cell RNA sequencing data demonstrated that *COX7B* was predominantly expressed in clusters 1, 3, 5, 6, and 8, while *NDUFA4* was expressed in clusters 1, 3, 5, 6, 7, and 9, corresponding to monocyte-macrophages, T cells, B cells, and platelets, respectively (Fig. 7B). Consequently, sepsis-related high transcript levels of *COX7B* and *NDUFA4* were primarily found in immune-related cells, with elevated transcription in monocyte-macrophages, T cells, and B cells (Fig. 7C-D).

## Discussion

Sepsis is a highly inflammatory condition that can result in septic shock and multi-organ dysfunction. Despite an in-depth understanding of sepsis pathogenesis, the mortality rate linked to this condition remains largely unchanged in both domestic and international clinical settings [22]. Since the 1970s, the significance of mitochondrial malfunction in the pathological process of sepsis has been a topic of debate. The observed effects may diverge due to variations in endotoxemia models (e.g., lipopolysaccharide [LPS] versus live bacteria, small animal versus large animal models) and differences in model generation (e.g., varying endotoxin or bacterial doses) [23]. Recent investigations utilizing clinical samples, coupled with more refined analytical techniques and robust study designs, have enhanced the reproducibility of mitochondrial function research. This advancement promises an enhanced comprehension of the causes behind mitochondrial dysfunction and its association with the severity and results of sepsis; concurrently, this understanding is vital for the forthcoming creation of treatments for sepsis, as sepsis continues to be a leading reason for death among patients in intensive care units. Mitochondria, double-membraned organelles ubiquitous in most eukaryotic cells, exhibit diameters ranging from 0.5 to 10 μm. Functionally, they serve as cellular powerhouses, generating energy through oxidative phosphorylation and synthesizing ATP. Perturbations in mitochondrial dynamics are intricately linked to various pathological conditions [24]. Although clinical use of antioxidants has demonstrated some benefit in sepsis management, therapeutic interventions targeting oxidative stress warrant careful consideration, as they may yield unintended consequences [25].

This study collected peripheral blood cells from patients with sepsis and healthy individuals for RNA sequencing in order to identify differentially expressed genes. Concurrently, MiAGs were obtained from the MitoCarta3.0 database. The intersection of differentially expressed genes and MiAGs was determined. We found that the GO enrichment analysis of crossover genes mainly involved intracellular membrane-bound organelles, especially mitochondria, in cellular components. The enrichment of molecular functions mainly involves catalytic activity and the binding of small molecules. Moreover, the biological processes associated with the intersecting genes primarily included small molecule metabolic process and organic acid metabolic process. Finally, core genes were selected using PPI analysis. Remarkably, the ten genes that were identified with transcript levels most closely correlated with sepsis (*NDUFA2, COX7C, UQCRH, UQCR11, NDUFB6, COX7B, NDUFA1, NDUFA4, COX7A2*, and *NDUFB3*) are all encoding subunits of the mitochondrial respiratory chain enzyme complexes. Of these genes, transcription levels of *COX7B* and *NDUFA4*, which showed a negative correlation with patient survival, had the greatest prognostic value. Both *COX7B* and *NDUFA4* are coding for subunits of cytochrome c oxidase (COX) [26]. Furthermore, we identified through single-cell sequencing that the high transcription levels of *COX7B* and *NDUFA4* related to sepsis are mainly found in immune-related cells, with increased transcription in monocyte macrophages, T cells, and B cells.

COX serves as the terminal enzyme complex of the mitochondrial respiratory chain, orchestrating the movement of electrons from the reduced form of cytochrome c towards oxygen. Disorders intertwined with *COX7B* encompass Linear Skin Defects with Multiple Congenital Anomalies [27]. Its associated pathways encompass respiratory electron transport, ATP synthesis coupled electron transport, and uncoupling protein-mediated heat production. In the oncological realm, investigations have unveiled a correlation between *COX7B* expression and infiltrative immune cells in esophageal carcinoma [27]. Within septic research, proteins within the COX family are essential. Throughout sepsis, inflammatory signals target COX, precipitating a marked inhibition of OXPHOS and an energy crisis [28]. Consequently, modulation of the OXPHOS process may emerge as a stratagem to ameliorate patient prognosis [29]. Recently, scholars have established an ex vivo sepsis model by inciting RAW264.7 macrophages with LPS, unearthing a heightened expression of *COX7B* in sepsis and its vital role in mitochondrial quality imbalance amid lipopolysaccharide-treated macrophages [30]. These findings underscore the potential research impetus of *COX7B* in sepsis. According to our research, *COX7B* is closely associated with the diagnosis and prognosis of sepsis. However, our research was limited by the small number of sequencing samples, which may lead to false positives. To address this limitation, we downloaded datasets from the GEO database pertaining to sepsis and normal control groups. These datasets were utilized for survival curve analysis, receiver operating characteristic (ROC) curve analysis, and meta-validation. Survival curve analysis revealed that patients with low expression of *COX7B* had higher 28-day survival rates compared to those with high expression, indicating a negative correlation between *COX7B* expression and sepsis patient survival, suggesting a close association between *COX7B* and sepsis prognosis. ROC curve analysis confirmed the high sensitivity and specificity of *COX7B*. Meta-analysis based on multiple GEO datasets revealed that *COX7B* was upregulated in the sepsis group while downregulated in the normal control group. These findings provide strong support for our study. In summation, our findings underscore the important diagnostic and prognostic utility of *COX7B* in sepsis.

*NDUFA4* (NADH: ubiquinone oxidoreductase subunit A4) was originally thought to be coding for a subunit of respiratory chain enzyme complex I but is now recognized as a bona fide subunit of COX [26]. In studies pertaining to head and neck paragangliomas (HNPGL), investigators employed lentivirus infection and puromycin screening to establish *NDUFA4* knockdown in PGL-626 cells. This experimental paradigm demonstrated the promotion of HNPGL progression by *NDUFA4*, while *NDUFA4* knockdown enhanced the inhibition of HNPGL progression mediated by metformin in a murine model [31]. Research associated with sepsis indicates a significant correlation between elevated expression of *NDUFA4* and poorer overall survival (OS) in patients with bacterial sepsis [32]. Another study suggests that miR-210-3p promotes myocardial cell apoptosis and mitochondrial dysfunction in sepsis-induced myocardial dysfunction by targeting the *NDUFA4* gene [33]. In our investigation, we observed a close association between *NDUFA4* and sepsis prognosis. Survival curves indicate that patients with low *NDUFA4* expression exhibit a higher 28-day survival rate compared to the high-expression group, correlating inversely with sepsis patient survival, consistent with prior research. To further comprehend *NDUFA4* expression across different populations, our meta-analysis results suggest upregulation of *NDUFA4* in the septic cohort and downregulation in the normal cohort. Of note, single-cell sequencing reveals predominant expression of *NDUFA4* in immune cells such as monocyte-macrophages, T cells, and B cells mitochondria. We posit that *NDUFA4* could serve as an innovative target for mitochondrial therapy, particularly in the realm of immunotherapy, thereby offering new avenues for clinical management of sepsis.

### Conclusions and limitations

This study screened and validated mitochondrial-related genes closely associated with sepsis through bioinformatics methods to identify mitochondrial-related genes significantly impacting the survival rates of sepsis patients. Our research provides valuable insights to guide subsequent functional studies on mitochondrial genes and their underlying mechanisms. However, a limitation of this study lies in its observational nature, as further functional validation of target genes is yet to be conducted. In addition, our study is limited by the low number of sequencing samples, which may lead to false positives. Furthermore, the inference is solely based on sequencing data, lacking sufficient feasibility, and should be further validated for its mechanism.

## Data Availability

We intend to share individual deidentified participant data. Peripheral blood RNA sequencing data from 20 patients with sepsis and 10 NC are available in the China National GeneBank DataBase (CNGBdb) and can be found below: https://db.cngb.org/, under the accession: CNP0002611, you can access it now and it’s valid forever. Our study used six datasets (GSE65682, GSE95233, GSE28750, GSE54514, GSE67652, and GSE69528), with all RNA sequencing data and clinical information coming from the GEO database.
